# Development of the scale on the effects of sleep disorder on stress: validity and reliability study

**DOI:** 10.1186/s12888-025-07099-2

**Published:** 2025-07-01

**Authors:** Arzu Karakaya, Guzel Nur Yıldız, Nuray Şimşek

**Affiliations:** 1 Institute of Health, Department of Psychiatric Mental Health Nursing, Erciyes University, Kayseri, 38039 Turkey; 2 Department of Therapy and Rehabilitation, Vocational School of Healt Services, Mus Alparslan University, Mus, Turkey; 3 Department of Dialysis, Vocational School of Health Services, Mus Alparslan University, Mus, Turkey; 4 Faculty of Health Sciences, Department of Psychiatric Mental Health Nursing, Erciyes University, Kayseri, 38039 Turkey

**Keywords:** Sleep disorder, Perceived stress, Cognitive theory, Scale development, Validity, Reliability

## Abstract

**Background:**

The relationship between sleep disorders and cognitive stress in clinical psychology, especially from the perspective of Beck's Cognitive Theory, holds critical importance. The current measurement tools do not adequately assess the cognitive components of sleep-related stress (e.g., automatic thoughts, cognitive distortions), highlighting the need for a new instrument in this field. Specifically, quantitatively evaluating the effects of insomnia on an individual's perception of the future, self-esteem, and worldview is fundamental for clinical interventions.

**Purpose:**

This study aims to develop a valid and reliable scale capable of measuring stress caused by sleep disorders with a multidisciplinary approach, based on Beck's Cognitive Theory's "cognitive triad" (perception of future/self/world) and the concept of cognitive distortion.

**Method:**

The study was conducted with 637 participants who had a Pittsburgh Sleep Quality Index (PSQI) score of ≥ 5. Data were collected using the Pittsburgh Sleep Quality Index, the Perceived Stress Scale, and a draft scale. Exploratory Factor Analysis (EFA) and Confirmatory Factor Analysis (CFA) were used to assess construct validity, while Cronbach's Alpha (*α*) and McDonald Omega (*ω*) were used for reliability analysis.

**Results:**

The results of the exploratory and confirmatory factor analyses revealed that the scale consists of two main dimensions: (1) Cognitive Distortions dimension (10 items, *α* = 0.952), which measures automatic negative thoughts such as "When I feel inadequate in any aspect due to sleep disorder" experienced by participants during sleep deprivation. (2) Cognitive Triad dimension (8 items, *α* = 0.925), which reflects disruptions in perceptions of the future, self, and the world (environment), with statements like "On days when I experience sleep disorders, I feel like my appearance looks bad." The overall reliability coefficients of the scale were found to be *α* = 0.964 and *ω* = 0.961. The developed scale was found to have a positive correlation with the Pittsburgh Sleep Quality Index, Perceived Stress Scale, and VAS.

**Conclusıons:**

The developed scale is the first tool to clinically and meaningfully reveal the cognitive stress mechanisms caused by sleep disorders. Its structure, directly aligning with the core components of Beck's Cognitive Theory, increases the potential for its use in cognitive-behavioral therapy protocols. Furthermore, the methodological framework it provides for cross-cultural validity studies lays the groundwork for comparative research on a global scale.

## Introduction

Sleep is a vital component for maintaining the health and well-being of individuals [21]. However, stress is a specific condition that arises when the emotional and physical limits of an organism are threatened and challenged, leading to physical and psychological symptoms. Stress triggers the immune response in the body and can lead to negative health outcomes such as inflammation. Prolonged stress reduces the body's capacity to manage inflammation, increasing the risk of susceptibility to various diseases [6].

### The importance of Beck’s cognitive theory in scale development

Everything created by a person is shaped through the individual's perceptual processes. According to Beck's cognitive theory (2006), stressful situations such as insomnia lead individuals to exaggerate negative outcomes, focus on threats, and underestimate their coping abilities. One of the core components of this theory is the tendency of individuals with insomnia to create catastrophe scenarios [3]. The process of catastrophizing involves irrational beliefs that future events will inevitably result in negative outcomes [16]. According to Beck, in perceived stressful situations, individuals tend to distort their thoughts [3]. Sleep disorders are situations where these catastrophic scenarios, which form the basis of these distortions, are produced [13]. These scenarios contain irrational beliefs that future events will have negative outcomes [31]. Individuals with insomnia tend to exaggerate the likelihood of negative effects, focus on negative potentials, and believe they will not be able to cope with these outcomes [15].

The process of individuals with sleep disorders producing catastrophe scenarios involves the following steps: First, the person becomes more focused on their sleep due to psychological arousal and starts monitoring their sleep. Then, they begin to create catastrophe scenarios about the negative outcomes of their sleep disorder, which increases the severity [29]. As a result, these negative thoughts further worsen the situation, and the destructive cycle of the sleep disorder continues. Research has shown that individuals with sleep disorders often exaggerate the harmful effects of the disorder on their functionality [9, 19]. In this context, the mechanism of mental distortions caused by sleep disorders is of great importance. Research is being conducted on how different types of sleep disorders (insomnia, obstructive sleep apnea, circadian rhythm disorders, etc.) transform cognitive perception. These changes are shaped not only by psychological factors but also by biological, developmental, and environmental influences. Individuals may sometimes interpret their experiences in a stubborn and inaccurate way [16]. In individuals with sleep disorders, there are significant differences in the way events are perceived and interpreted. This leads to different effects on individuals from the same event and causes interpersonal reactions to vary. Within this theoretical framework, in order to better understand the complex relationship between sleep disorders and stress, it is necessary to focus on how individuals respond to sleep disorders in the light of Beck's cognitive theory and how these responses affect their stress levels.

### Bidirectional relationship between sleep disorders and stress

A literature review indicates that the close relationship between stress and sleep is well known [35]. Stress can lead to insomnia, and insomnia itself can be a result of stress [14]. Other sleep disorders such as hypersomnia and obstructive sleep apnea can develop as a result of autonomic nervous system dysfunction, physical problems (tumors, head trauma), medical conditions (multiple sclerosis, depression, obesity), or substance abuse (drugs and alcohol) [23]. Specifically, obstructive sleep apnea has been shown to hinder restorative sleep, leading to increased stress levels and resulting in higher levels of the stress hormone cortisol [5]. Additionally, it has been found that stress can lead to unhealthy habits (smoking, alcohol abuse, excessive caffeine consumption, overeating, and lack of exercise), which in turn increases the risk of sleep apnea [30]. Overall, it has been demonstrated that each type of sleep disorder serves as a source of stress, creating a continuous cycle that negatively impacts physical and psychological health [11, 12]. This bidirectional relationship plays a significant role in mechanisms that help maintain the body's homeostasis in response to internal or external challenges [22]. Various animal and human studies have shown that stress-inducing factors can affect the wake-sleep cycle in different ways, with this effect being significantly determined by the type of stressor, the duration of exposure (acute or chronic), and individual differences [17]. However, it should be noted that research on the clarity of the relationship between sleep and stress is still ongoing. The primary focus of this study is to measure the unidirectional effect of sleep disorders on perceived stress levels. Beck's cognitive theory contributes to understanding this mechanism by explaining how sleep deprivation shapes cognitive distortions (catastrophizing, exaggerated perception of threat).

The literature emphasizes the presence of multidimensional factors, such as physical activity, chronic diseases, and particularly sleep disorders, that affect stress levels [20, 35]. However, there is a notable lack of a psychometrically valid and reliable instrument that directly measures the impact of sleep disorders on individuals'perceived stress levels. Currently, the heterogeneous nature of stress-inducing factors makes it difficult to identify the areas in which individuals are most vulnerable, preventing clinical interventions from being designed with a targeted focus.

In this context, existing scales examining the sleep-stress relationship [10, 27, 34] generally focus on measuring overall stress levels and fail to quantify the specific stress caused by disruptions in sleep quality. The primary aim of our study is to develop a valid and reliable scale, based on Beck's Cognitive Theory's"cognitive triad"(perception of future/self/world) and the concept of cognitive distortion, that can measure stress caused by sleep disorders with a multidisciplinary approach. This initiative will not only illuminate the mechanisms of the etiological relationship but also provide a dynamic metric for assessing the effectiveness of cognitive-behavioral therapy and pharmacological interventions.

## Method

### Type of study

The study is a scale development study that includes the validity and reliability stages of the methodological research type.

### Location and date of the study

The data of the study were collected between 16.05.2023 and 30.06–2023 at Muş State Hospital Sleep Clinic and Neurology Clinic. This scale was developed for individuals over the age of 18 who have been diagnosed with sleep disorders. An average of 50–60 people aged 18 and over apply to the Sleep Clinic and Neurology Clinic daily. It is recommended in scale development studies that the data be collected from two different samples for Exploratory Factor Analysis (EFA) and Confirmatory Factor Analysis (CFA). For this reason, the data collection was performed in two stages.

### Population and sample of the study

The population of the study consisted of the patients who applied to the Sleep Clinic between 16 May and 30 June 2024. The Pittsburgh Sleep Quality Index (PSQI) is a reliable and valid measurement tool that is widely accepted worldwide in determining and rating sleep disorders. PSQI does not limit sleep quality to a general assessment, but examines various dimensions of sleep disorders in detail, and accurately measures individuals'sleep habits, duration, frequency and general sleep disorder levels. Thanks to these features, PSQI is extremely effective in diagnosing sleep disorders and correctly distinguishing patient groups. In addition, the reliable results provided by the use of this scale in numerous clinical and psychological studies clearly demonstrate its adequacy in distinguishing individuals with sleep disorders. Therefore, in this study, patients with sleep disorders were distinguished using the PSQI. The sample of the study consisted of those who met the inclusion criteria.

### Inclusion criteria

- Being over 18 years old,
- Having a score of 5 or above on the PSQI,
- Having no communication problems,
- Confirmation of sleep problems,
- Completing the Voluntary Informed Consent Form.

### Exclusion criteria

- Having a score of 4 or less on the PSQI,
- Incomplete data
- Diagnosis of a physical or mental illness that might prevent participation in the study,
-Taking medication related to sleep disorders,
-Being addicted to alcohol and drugs,
-Participants'lack of language knowledge at a level sufficient to understand the survey and tests constitutes an exclusion criterion.

In the first stage, the data were collected from *n* = 392 people, and *n*** = **300 people in the second stage. A total of 55 people were excluded from the study because the *PSQI* score of *n* = 27 people in the first stage and *n* = 28 people in the second stage was below 5. The sample of the study consisted of *n* = 637 people.

### The use of cognitive theory in scale development

Beck's cognitive theory serves as the conceptual framework, item formulation, and structural validity foundation in the development of a measurement tool for sleep-related stress. Here are the roles of the two main components of the theory in the scale development process:

1. Cognitive triad
  Beck's concept of the"cognitive triad"suggests that individuals have systematic distortions in their perceptions of the self, the future, and the environment [3]. In the scale, this triad is represented as follows:
  Self-perception:
    Statements like"When I feel that my appearance looks bad due to my sleep disorder"measure negative beliefs about the individual’s coping capacity.
  Expectations for the future:
    Statements such as"When I lose hope for the future due to my sleep disorder"or"When I lose interest in life because of my sleep disorder"reflect a tendency for catastrophizing [16].
  Interpretation of the world (environment):
    Items like"When I feel that I cannot meet the needs of my family or those around me due to my sleep disorder"assess perceived negativity in social relationships.
    These dimensions, confirmed through factor analysis, support the structural validity of the scale [27].
2. Types of cognitive distortions

The cognitive distortions defined by Beck are directly reflected in the content of the scale items:

**Table:** 

Distortion Type	Scale Item Example	Theoretical Basis
Selective Abstraction	*"When I am unable to express myself in interpersonal relationships due to my sleep disorder"*	Exaggeration of temporary problems [3]
Overgeneralization	*"When I feel that my quality of life has decreased because of my sleep disorder"*	Drawing universal conclusions from a single event [13]
Automatic Thought	*"When I struggle to cope with interpersonal communication issues due to my sleep disorder"*	Rejection of grey areas [29]
Catastrophizing	*"When I feel inadequate in any area due to my sleep disorder"*	Exaggeration of probabilities [15]

These distortions have been used to ensure content validity in alignment with the DSM-5 criteria for sleep disorders and insomnia.

### The methodological ıntegration of the theoretical model

1. Item pool creation:
  18 items were determined using patient interviews, expert opinions, and Beck's list of cognitive distortions.
  Example:"When I experience physical symptoms (nausea, diarrhea, constipation, headache, etc.) due to my sleep disorder"represents the distortion of concretization.
2. Determining the factor structure:
  In exploratory factor analysis (EFA), 3 main components were obtained, in line with the cognitive triad.

Beck's cognitive theory has served as a critical guide in mapping the cognitive interaction between sleep and stress and translating these dynamics into scale items. This approach allows for targeting clinical interventions by quantifying not only the severity of symptoms but also the underlying mental processes.

### Data collection tools

The data collection tools of the study were the “Personal Information Form”, “Pittsburgh Sleep Quality Index”, “Perceived Stress Scale”, and “Visual Analog Scale” to determine the effects of sleep disorder on stress levels and the “The Scale on The Effects of Sleep Disorder on Stress” (SESDS) in draft form. The data were collected face to face by making explanations to the individuals. The data collection process took 10–15 min.

#### Personal information form

The form included questions on the participants’ gender, age, etc.

#### Pittsburgh Sleep Quality Index (PSQI)

The Turkish validity and reliability study of the scale, which was developed by Buysse et al. in 1989, was conducted by Ağargün et al. in 1996 [1, 4]. The scale consists of 24 questions, the first of which are self-report assessment questions answered by the individual. The last 5 questions are answered by the individual’s spouse/roommate. The scale consists of 7 components (“Subjective sleep quality, Sleep latency, Sleep duration, Habitual sleep efficiency, Sleep disorder, Sleeping medication use, and Daytime dysfunction”). Scores between 0 and 21 can be obtained from the scale (7 components are scored between 0–3). The sum of 7 components gives the final result. Scores below 5 indicate normal sleep quality and scores of 5 and above indicate that the individual has the sleep disorder. An increase in the PSQI score is an indicator of the severity of an individual's sleep disorder. The PSQI was used in this study to identify individuals with sleep disorders. Individuals with scores below 5 were excluded from the study. Thus, patients with sleep disorders were included in the study. Ağargün et al. reported that the Cronbach Alpha value of the scale was 0.804 [1, 4]. The Cronbach Alpha value of the scale was found to be 0.725 in this study.

#### Perceived Stress Scale (PSS)

PSS was developed by Cohen et al. in 1983. The scale was adapted into Turkish by Eskin et al. in 2013. The scale consists of two dimensions:"Perceived insufficient self-efficacy"and"Perceived stress/distress". The scale consists of 10 questions. It has a 5-point Likert style (0 = never and 4 = very often). The lowest score that can be obtained from the scale is “0” and the highest score is “40”. An increase in score indicates that the individual’s perception of stress increases. The use of PSS allows for a more accurate assessment of the effects of sleep disorders on stress by objectively assessing participants'perceptions of stress. In addition, the short and understandable structure of PSS allows for quick and easy measurement of stress levels. Therefore, the use of PSS in the study plays an important role in understanding the dynamic relationship between stress and sleep disorders and increases the validity of the scale. Eskin et al. reported that the Cronbach Alpha value of the scale was 0.82. The Cronbach Alpha value was determined to be 0.66 in this study.

#### Visual Analog Scale for determining the effects of sleep disorder on stress levels (VAS)

It was found in the literature review that VAS was used for many purposes. For this reason, VAS was used to self-report individuals’ stress levels caused by sleep disorders in this study. The"VAS for Determining the Effects of Sleep Disorder on Stress Levels"used in the study is a very practical tool for quickly and subjectively assessing individuals'stress levels. VAS allows participants to easily express their personal stress perceptions by offering the opportunity to evaluate a subjective experience such as stress on a numerical scale. In this study, which aims to develop a scale to determine the effects of sleep disorders on stress, the use of VAS allows for direct and rapid measurement of the intensity and change of stress. In addition, the simple and user-friendly structure of VAS allows participants to have less difficulty in responding and ensures that the survey provides more accurate results. The short-term and open-ended structure of the scale increases the time efficiency of the study, while at the same time offering great flexibility in reflecting the participants'subjective experiences of the effects of sleep disorders on stress. Therefore, VAS supports the reliability and validity of the study as a very effective tool in determining the effects of sleep disorders on stress levels. Individuals were asked how much sleep disorder affected their stress levels and were asked to mark the level between 0–10.

#### The Scale on the Effects of Sleep Disorder on Stress (SESDS)

The scale was developed in 3 stages.

#### First stage (Creating the item pool and making it ready for implementation)

The item pool was created for the development of the SESDS in the first stage. The item pool was created by considering Beck’s “Cognitive Theory”. To create the item pool, 7 patients were interviewed about their sleep problems and the stress levels they experienced during the day. In this study, purposive sampling method was used in developing the scale items. Seven patients were selected from individuals who had sleep disorders and stress-related experiences. Scale items were determined in line with the interviews conducted with the participants in order to better understand their perceptions of the effects of sleep disorders on stress. These interviews aimed to ensure the content validity of the scale by utilizing the participants'personal experiences and feedback. The items that emerged as a result of these interviews were shaped to accurately measure the effects of sleep disorders on stress levels in accordance with the purpose of the study. In this study, the existing literature was thoroughly reviewed in order to understand the relationship between sleep disorders and stress. In the literature review, studies addressing the effects of sleep disorders on the psychological and physiological health of individuals, studies examining the effects of stress on sleep, and previous studies on the interaction of both factors were comprehensively reviewed. In addition, studies conducted to understand how sleep disorders and stress differ in various clinical populations and the effects of these relationships at the individual level were also evaluated. In addition, studies on theories were conducted in order to base this scale, which was developed to determine the effect of sleep disorders on stress levels, on a theoretical substructure. Beck's cognitive model, which was considered appropriate by the researchers, was selected to explain the effect of sleep disorders on stress. This literature review provided a theoretical basis for us to better understand the effect of sleep disorders on stress and guided the scale development process. As a result of interviews and literature reviews, an item pool of 27 items was created. The answer options of the scale were prepared in a 5-point Likert style (“1 = I don't feel stressed at all” and “5 = I feel very stressed”). The item pool was sent to 15 experts (7 experts in nursing principles, 1 expert in internal medicine nursing, 4 experts in psychiatric diseases nursing, 1 expert in surgical diseases nursing, and 2 experts in Turkish Language and Literature). The Content Validity Index (CVI) was calculated in line with expert opinions. Two items were removed from the item pool because their CVI was below 0.80 and 25 items had a CVI value above 0.80 on an item basis. The pre-implementation stage started with 25 items.

#### Second stage (Pilot implementation)

Preliminary implementation was conducted with 10 people, who were asked to evaluate the items in terms of readability and understandability. The main implementation stage was completed with 25 items.

#### Third stage (Main implementation)

The data collection stage for the development of SESDS was performed in two stages. In the first stage, 392 people were reached and EFA and reliability analysis were performed. A total of 300 people were reached in the second stage and CFA was performed. Data were collected from different samples for EFA and CFA. Care was taken to ensure that patients participating in the EFA phase were not included in the CFA phase.

### Statistical analysis

Data was analyzed using SPSS package program and AMOS package program. Frequency, percentage, mean score and standard deviation values were used in statistical analysis. Suitability of the dataset for analysis and sample adequacy were determined by Kaiser Meyer Olkin (KMO) test (> *0.60*), Significant Bartlett’s Sphericity Test (< *0.05*), and Anti-Image Correlation Values (> *0.50*). Content validity, construct validity, and criterion validity methods were used to ensure validity. Items with values above *0.80* on the CVI were added to the item pool. EFA were used to ensure construct validity. In the EFA, the Varimax Rotation method, which is the most preferred orthogonal rotation method in scale development studies, was used. In the EFA, overlapping items were removed. An overlapping item is the situation where the items of the scale are very similar to each other or are questions that measure the same concept. If an item has loadings in more than one dimension; if the difference between the two highest loadings is below 0.10, this item is considered overlapping, and it is recommended to be removed from the data set. CFA was performed to test and verify the suitability of the factor structure of the obtained scale. “Relative Chi Square Index (CMIN/DF), *x*^*2*^* p*, Comparative Fit Index (CFI), Root Mean Square Error of Approximation (RMSEA), Root Mean Square Residual (RMR), Standardized Root Mean Square Residual (SRMR), Normed Fit Index (NFI), Trucker-Lewis Index (TLI), Incremental Fit Index (IFI), Parsimonious Goodness of Fit Index (PGFI), and Parsimony Normed Fit Index (PNFI)” Fit Index Values were used in the CFA [2, 18]. The Parallel Forms Method was used to ensure criterion validity. Criterion validity is an important type of validity that evaluates how accurately a test or scale measures by determining how compatible it is with a reliable and valid external criterion. In this study, VAS, Pittsburgh Sleep Quality Index, Perceived Stress Scale were used to ensure the criterion validity of the scale. In this study, the significance of the correlation analysis between the developed scale and the PSS and VAS used as parallel forms shows that the developed scale meets the criterion validity. Cronbach Alpha Values, McDonald Omega Coefficient, and Split-Half Reliability Coefficient were used to determine reliability. The lowest acceptable value for Cronbach's Alpha value and McDonald's Omega value is 0.60. As the Cronbach's Alpha value and McDonald's Omega value approach 1, the reliability of the scale increases. In this study, in order to test the discriminative power of the scale, participants were divided into the lower 27% (low scores) and upper 27% (high scores) quartiles. The scale score averages of both groups were calculated and the difference between them was examined with an independent sample t-test. The statistically significant difference between the score averages of the lower and upper quartiles shows that the scale can successfully distinguish individuals at different levels and therefore is sufficient in terms of discriminative validity [8, 25, 28].

## Results

### Characteristics of participants

In the first stage of the study, it was found that 76.2% of the patients who participated in the first stage were women, 84.7% did not have any chronic diseases, and 88.8% were between the ages of 18–25. Participants were asked to rate the impact of their sleep disturbance levels on their stress levels; the mean was found to be 6.88 ± 2.68, with “0” being the lowest and “10” being the highest (Table 1).

**Table 1:** Distribution of participants according to sociodemographic characteristics (*N* = 637)

		**EFA(*n* = *365*)**		**CFA (*n* = *272*)**	
**Features**	**Variables**	***n***	**%**	***n***	**%**
**Gender**	Woman	278	76.2	171	62.9
	Men	87	23.8	101	37.1
**Chronic disease**	Yes	56	15.3	115	42.3
	No	309	84.7	157	57.7
**Age**	18–25	324	88.8	118	43.4
	26 and over	41	11.2	154	56.6
**Visual Analog Scale for Determining the Effects of Sleep Disorder on Stress Levels**		6.88 ± 2.68 (Min = 0, Max = 10)		7.52 ± 2.39 (Min = 0, Max = 10)	

*n* = frequency, % = percentage

In the second stage of the study, it was found that 62.9% of the patients who participated in the second stage were women, 57.7% did not have any chronic diseases, and 56.6% were 26 years of age or older. Participants were asked to rate the effects of their sleep disturbance levels on their stress levels; the average was determined as 7.52 ± 2.39, with “0” being the lowest and “10” being the highest (Table 1).

### Findings regarding validity

1. Exploratory Factor Analysis (EFA)

Before the EFA, item correlation values of the items, Cronbach Alpha Values (if the item was deleted), KMO values, Bartlett’s Sphericity Test results, and normality distributions were examined and it was found that the item total correlation value of the items varied between 0.671 and 0.853, the Cronbach Alpha value of 25 items was 0.976, the KMO value was 0.971, the Bartlett’s Test of Sphericity Value was 8887.068 (*p* < 0.001), and the data were normally distributed. Item correlation values were above 0.30. EFA was started with 25 Items. The Vertical Rotation (Varimax) Method was preferred in EFA (Table 2).

**Table 2:** Mean, standard deviation, total correlation of the items, and Cronbach α values if the item was deleted

	**Mean**	**Standard Deviation**	**Item Total Correlation Value**	**Cronbach Alpha Value if the Item was Deleted**
i1	2.96	1.14	.719	.976
i2	3.10	1.17	.769	.975
i3	2.96	1.21	.773	.975
i4	2.87	1.29	.806	.975
i5	3.20	1.27	.765	.975
i6	3.16	1.29	.807	.975
i7	3.17	1.27	.833	.975
i8	2.88	1.33	.847	.975
i9	3.03	1.31	.825	.975
i10	2.70	1.35	.735	.976
i11	3.09	1.35	.823	.975
i12	2.79	1.41	.781	.975
i13	3.13	1.32	.853	.975
i14	3.06	1.36	.849	.975
i15	2.91	1.32	.786	.975
i16	2.90	1.43	.770	.975
i17	3.01	1.35	.748	.976
i18	2.68	1.32	.713	.976
i19	2.84	1.36	.780	.975
i20	2.55	1.35	.671	.976
i21	2.94	1.40	.761	.975
i22	3.12	1.31	.796	.975
i23	2.96	1.37	.710	.976
i24	2.92	1.32	.828	.975
i25	3.19	1.40	.764	.975

Since 7 items were overlapping items, they were removed from the analyses (*i₁₇*, *i₁₄*, *i₂₄*, *i₂₅*, *i₂₂*, *i₁₃*, and *i₁₀*, respectively). It was found that the explained variance rate increased when items were removed. The results of the KMO Test, Bartlett’s Sphericity Test, and Anti-Image Correlation Values of 18 items were sufficient (KMO = 0.963, Bartlett’s Sphericity Test, *p* = 0.001, Anti-Image Correlation = 0.936–0.980). The results of the EFA that was conducted with 18 items are given in Table 3 (Table 3).

**Table 3:** Scale EFA results

Items	Cognitive Theory	Common Factor Variance	Factors	
			**1**	**2**
7. When I feel that my quality of life has decreased because of my sleep disorder	Cognitive Distortions	0.781	0.795	
2. When I feel like a failure because of the sleep disorder	Cognitive Distortions	0.712	0.777	
5. When I wake up feeling tired because of the sleep disorder	Cognitive Distortions	0.702	0.776	
9. When I feel tense because of the sleep disorder	Cognitive Distortions	0.752	0.772	
6. When I cannot do my daily work because of the sleep disorder	Cognitive Distortions	0.720	0.752	
4. When I cannot express myself in interpersonal relationships because of the sleep disorder	Cognitive Distortions	0.733	0.746	
3. When I feel inadequate about anything because of the sleep disorder	Cognitive Distortions	0.692	0.737	
8. When I have difficulty coping with my interpersonal communication problems caused by the sleep disorder	Cognitive Distortions	0.733	0.737	
1. When I cannot focus on my work because of the sleep disorder	Cognitive Distortions	0.637	0.734	
11. When I feel angry because of the sleep disorder	Cognitive Distortions	0.704	0.678	
18. When I have nutrition problems because of the sleep disorder	Cognitive Triad	0.681		0.775
20. When I feel unable to focus on daily activities such as reading the newspaper or watching television because of the sleep disorder	Cognitive Triad	0.650		0.764
16. When my hope for the future decreases because of the sleep disorder	Cognitive Triad	0.730		0.760
19. When I feel like I am not adequate for my family or those around me because of the sleep disorder	Cognitive Triad	0.737		0.760
15. When my interest in life decreases because of my sleep disorder	Cognitive Triad	0.695		0.686
12. When I feel lonely because of the sleep disorder	Cognitive Triad	0.674		0.629
21. When I experience physical symptoms (nausea, diarrhea, constipation, headache, etc.) because of the sleep disorder	Cognitive Triad	0.627		0.628
23. When I feel like my appearance looks bad on days when I have a sleep disorder	Cognitive Triad	0.541		0.578
Eigenvalue (Total = 12.546)			11.497	1.049
Variance explained (Total = 69.701%)			63.872	5.830

**Fig. 1:**
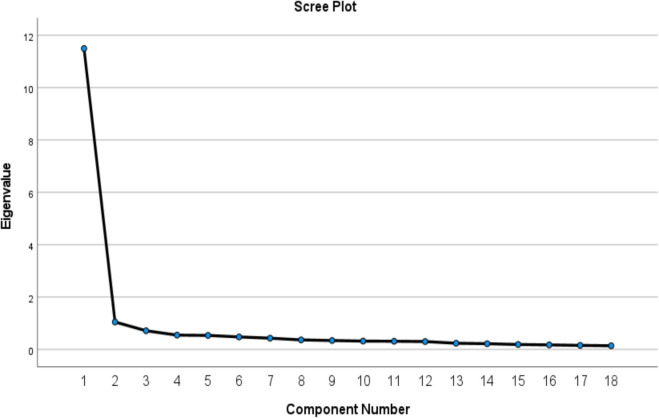
Scree plot chart factor structure

The analysis results and scree plots showed that the scale items were grouped under 2 factors. The presence of 2 components with eigenvalues above 1 indicated that the scale had a 2-factor structure (Table 3) (Fig. 1).

It was found that the 1 st factor consisted of items *i₁*, *i₂*, *i₃*, *i₄*, *i₅*, *i₆*, *i₇*, *i₈*, *i₉*, and *i₁₁* (10 items) and explained 63.872% of the total variance and consisted of items related to the concepts of “Cognitive Distortions” according to Beck’s Cognitive Theory. For this reason, this dimension was called “Cognitive Distortions” (Table 3).

Since the 2nd factor consisted of items *i₁₂, i₁₅, i₁₆, i₁₈, i₁₉, i₂₀, i₂₁,* and *i₂₃*, (8 items) and 5.83% of the total variance consisted of items containing the concepts of “Cognitive Triad” according to Beck’s Cognitive Theory, this dimension was called “Cognitive Triad” (Table 3).

When the 18-item scale was examined as a whole, it was seen that it had a 2-factor structure and explained 69.701% of the total variance (Table 3). The scale explained sufficient variance. The obtained values showed that the scale was sufficient to determine individuals’ stress levels because of sleep disorders.

2. Confirmatory Factor Analysis (CFA)

**Fig. 2:**
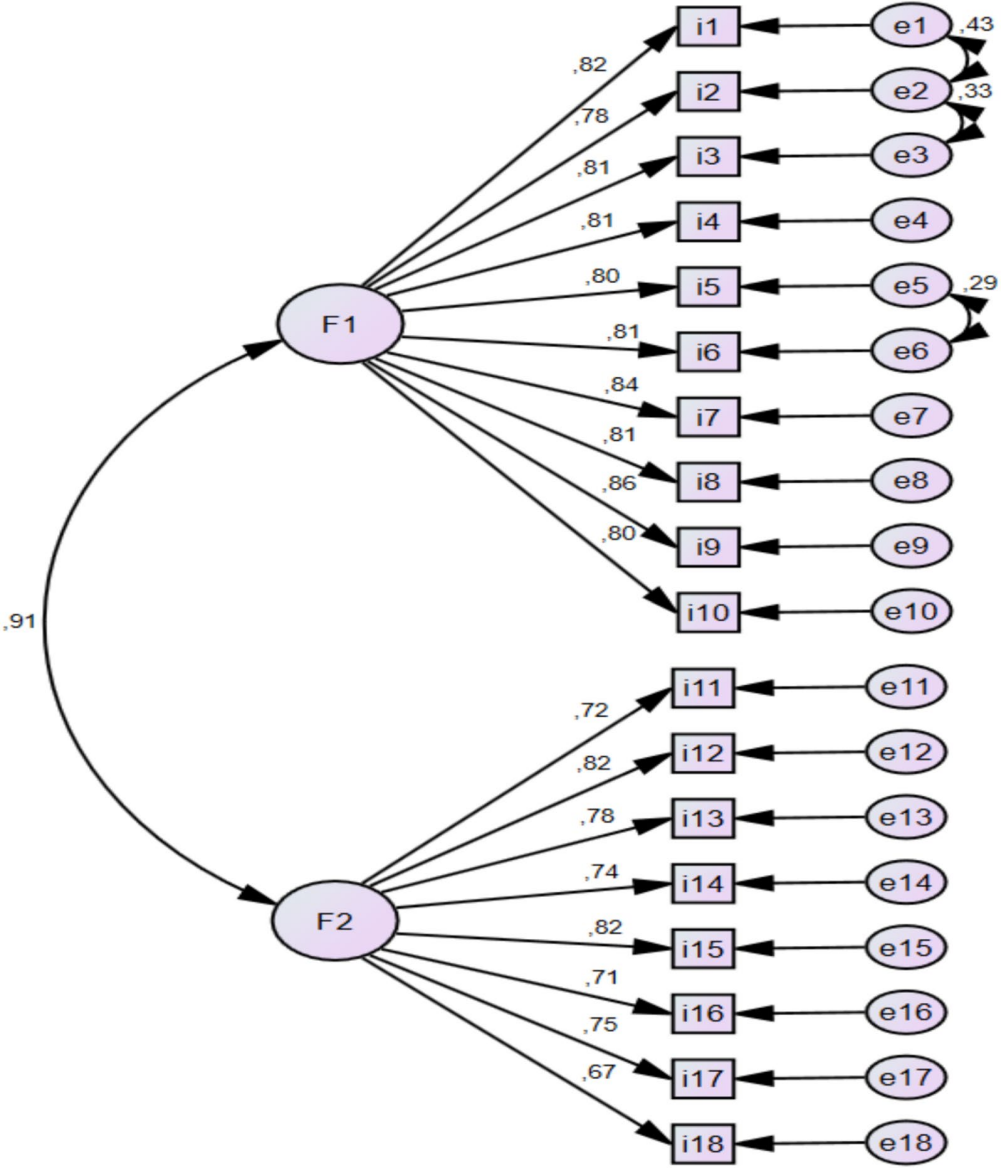
Confirmatory factor analysis diagram. CMIN/DF = 2.001, *x*.^*2*^*p* = 0.001, CFI = 0.946, RMSEA = 0.078, RMR = 0.051, SRMR = 0.0370, NFI = 0.916, TLI = 0.937, IFI = 0.946, PGFI = 0.673, PNFI = 0.784

CFA was used with the AMOS Package Program. The data were collected from 300 patients for CFA. However, 28 patients were excluded from the dataset because their PSQI was 4 or less. CFA was performed with analyses obtained from 272 people. A total of 3 suitable modification suggestions (*i1-i2, i2-i3, i5-i6*) were found in the AMOS package program (Fig. 2). Fit values were evaluated considering the literature data and the obtained fit indices were found to be fit (*CMIN/DF* = 2.001, *x*^*2*^*p* = 0.001, *CFI* = 0.946, *RMSEA* = 0.078, *RMR* = 0.051, *SRMR* = 0.0370, *NFI* = 0.916, *TLI* = 0.937, *IFI* = 0.946, *PGFI* = 0.673, *PNFI* = 0.784) [2, 18].

It was found in the analysis conducted as a result of CFA that the 10 items in the 1 st factor had standard solutions ranging between 0.78–0.86, and the 8 items in the 2nd factor had standard solutions ranging between 0.67–0.82 (Fig. 2). The items were important for the factors in which they were found. The Path Diagram was examined and it was found that the obtained values were appropriate in terms of item-factor fit (Fig. 2).

### Findings regarding reliability

1. Internal consistency (Cronbach Alpha) and McDonald’s omega coefficients

Cronbach’s alpha coefficient was calculated to determine the reliability of the scale and it was found that the *α* value of the factor “Cognitive Distortions” (F1) was 0.952, and the *α* value of the factor “Cognitive Triad” (F2) was 0.925. The ***α*** value for the entire scale was determined to be 0.964.

To determine the reliability of the scale, McDonald’s omega coefficient was calculated (*ω* = 0.957 for “Cognitive Distortions” (F1) and *ω* = 0.926 for “Cognitive Triad” (F2). McDonald’s omega for the whole scale was determined to be 0.964. These values showed that the scale was quite reliable [8, 25].

2. Split-half reliability coefficient number

As a result of the Split-Half Reliability Analysis of the final form of the scale, which consisted of 18 items, it was found that the Spearman-Brown Correlation Value (*r* = 0.921) and the Guttmann Split-Half Coefficient Value (*r* = 0.919) had sufficient values. The Cronbach Alpha Value of the 1 st part of the scale was 0.928, and the Cronbach Alpha Value of the 2nd part was 0.950. The Split-Half Reliability Analysis results showed that the scale was reliable [28].

3. Determining the discrimination of the scale

A statistically significant difference was detected between the SESDS and sub-dimension score averages of the lower 27% and upper 27% groups (*p* < 0.05). This result shows that the scale can successfully distinguish individuals at different levels and that the discriminant validity of the scale is sufficient (Table 4).

4. Relationship between VAS, Pittsburgh sleep quality index, perceived stress scale and the effects of sleep disorder on stress scale

**Table 4:** Discrimination power of the scale, sub-dimensions, and items

Items		**Mean**	**Standard Deviation**	**Test and significant**
i1	1 st Quarter	2.27	.790	***t* = −14.061** ***p* < 0.001**
	2nd Quarter	3.62	1.03	
i2	1 st Quarter	2.36	.836	***t* = −15.207** ***p* < 0.001**
	2nd Quarter	3.82	.994	
i3	1 st Quarter	2.12	.789	***t* = −17.972** ***p* < 0.001**
	2nd Quarter	3.78	.971	
i4	1 st Quarter	1.96	.768	***t* = −18.704** ***p* < 0.001**
	2nd Quarter	3.76	1.05	
i5	1 st Quarter	2.32	.869	***t* = −17.734** ***p* < 0.001**
	2nd Quarter	4.05	.985	
i6	1 st Quarter	2.23	.946	***t* = −19.014** ***p* < 0.001**
	2nd Quarter	4.05	.883	
i7	1 st Quarter	2.21	.803	***t* = −21.959** ***p* < 0.001**
	2nd Quarter	4.11	.855	
i8	1 st Quarter	1.84	.708	***t* = −22.780** ***p* < 0.001**
	2nd Quarter	3.88	.982	
i9	1 st Quarter	2.03	.780	***t* = −22.225** ***p* < 0.001**
	2nd Quarter	4.01	.921	
i11	1 st Quarter	2.03	.815	***t* = −23.669** ***p* < 0.001**
	2nd Quarter	4.13	.881	
i12	1 st Quarter	1.77	.777	***t* = −19.802** ***p* < 0.001**
	2nd Quarter	3.79	1.14	
i15	1 st Quarter	1.96	.824	***t* = −19.547** ***p* < 0.001**
	2nd Quarter	3.84	1.01	
i16	1 st Quarter	1.91	.932	***t* = −18.191** ***p* < 0.001**
	2nd Quarter	3.87	1.13	
i18	1 st Quarter	1.84	.838	***t* = −15.255** ***p* < 0.001**
	2nd Quarter	3.49	1.198	
i19	1 st Quarter	1.86	.789	***t* = −19.205** ***p* < 0.001**
	2nd Quarter	3.78	1.10	
i20	1 st Quarter	1.76	.814	***t* = −13.442** ***p* < 0.001**
	2nd Quarter	3.31	1.33	
i21	1 st Quarter	1.99	.925	***t* =—17.205** ***p* < 0.001**
	2nd Quarter	3.86	1.14	
i23	1 st Quarter	2.14	1.01	**t = −13.753** ***p* < 0.001**
	2nd Quarter	3.75	1.21	
Cognitive Distortions (f1)	1 st Quarter	2.14	.52	**t = −28.827** **p < 0.001**
	2nd Quarter	3.92	.65	
Cognitive Distortions (f2)	1 st Quarter	1.90	.55	***t* = −26.896** ***p* < 0.001**
	2nd Quarter	3.71	.72	
SESDS	1 st Quarter	2.03	.46	***t* = −32.151** **p < 0.001**
	2nd Quarter	3.83	.60	

*i* items, *SESDS* The Scale on The Effects of Sleep Disorder on Stress, 1 st Quarter = Lowest 27%, 2nd Quarter = The highest 27%

The scale and all sub-dimensions showed a strong and positive correlation with each other. A positive and moderate relationship was detected between the SESDS and its sub-dimensions and the PSS, PSQI, and VAS scores. These findings show that the scale is reliable and criterion validity is achieved (Table 5).

5. Relationship between SESDS and sub-dimensions of items

**Table 5:** Average scores for SESDS and sub-dimensions, minimum and maximum scores, and correlation analysis results

	**Mean ± sd** **(min–max)**		**F1**	**F2**	**SESDS**
**Cognitive Distortions (f1)**	2.95 ± 1.08 (Min = 1, Max = 5)	*r*	**1**	**.842**	**.967**
		*p*	**.**	**.001**	**.001**
**Cognitive Distortions (f2)**	2.70 ± 1.11 (Min = 1, Max = 5)	*r*	**.842**	**1**	**.951**
		*p*	**.001**	**.**	**.001**
**SESDS**	2.84 ± 1.05 (Min = 1, Max = 5)	*r*	**.968**	**.953**	**1**
		*p*	**.001**	**.001**	**.**
**PSQI**	9.60 ± 3.13 (Min = 5, Max = 18)	*r*	**.371**	**.377**	**.389**
		*p*	**.001**	**.001**	**.001**
**PSS**	21.49 ± 5.76 (5–38)	*r*	**.439**	**.355**	**.420**
		*p*	**.001**	**.001**	**.001**
**VAS**	6.88 ± 2.68 (Min = 0, Max = 10)	*r*	**.430**	**.387**	**.426**
		*p*	**.001**	**.001**	**.001**

*SESDS* The Scale on The Effects of Sleep Disorder on Stress, *PSQI* Pittsburgh Sleep Quality Index, *PSS* Perceived Stress Scale, *VAS* Visual Analog Scale, *r* correlation, *p* significant, *f* factor

It was determined that there was a positive, strong and statistically significant relationship between the items and SESDS and its sub-dimensions. This finding shows that the items are significantly related to the scale and its sub-dimensions (Table 6).

**Table 6:** Relationship between SESDS and sub-dimensions of items

		Cognitive Distortions (f1)	Cognitive Distortions (f2)	SESDS
i1	*r*	0.785	0.651	0.754
	*p*	0.001	0.001	0.001
i2	*r*	0.831	0.684	0.796
	*p*	0.001	0.001	0.001
i3	*r*	0.829	0.703	0.804
	*p*	0.001	0.001	0.001
i4	*r*	0.857	0.735	0.835
	*p*	0.001	0.001	0.001
i5	*r*	0.834	0.666	0.789
	*p*	0.001	0.001	0.001
i6	*r*	0.853	0.712	0.822
	*p*	0.001	0.001	0.001
i7	*r*	0.887	0.727	0.849
	*p*	0.001	0.001	0.001
i8	*r*	0.884	0.776	0.870
	*p*	0.001	0.001	0.001
i9	*r*	0.873	0.723	0.839
	*p*	0.001	0.001	0.001
i10	*r*	0.699	0.726	0.741
	*p*	0.001	0.001	0.001
i11	*r*	0.844	0.749	0.834
	*p*	0.001	0.001	0.001
i12	*r*	0.750	0.819	0.814
	*p*	0.001	0.001	0.001
i13	*r*	0.822	0.792	0.842
	*p*	0.001	0.001	0.001
i14	*r*	0.812	0.803	0.842
	*p*	0.001	0.001	0.001
i15	*r*	0.731	0.831	0.809
	*p*	0.001	0.001	0.001
i16	*r*	0.692	0.855	0.798
	*p*	0.001	0.001	0.001
i17	*r*	0.715	0.716	0.745
	*p*	0.001	0.001	0.001
i18	*r*	0.620	0.796	0.729
	*p*	0.001	0.001	0.001

*r* Corelation, *p* Significant, *SESDS* The Scale on The Effects of Sleep Disorder on Stress

## Discussion

The study was conducted to develop the “The Scale on The Effects of Sleep Disorder on Stress”. The findings obtained from the study were discussed in line with the literature data.

### Content validity

To calculate the content validity of the scale, a pool of items was sent to 15 experts, and the item-based Content Validity Index (CVI) was calculated. Two items were removed from the item pool because their Content Validity Index was below 0.80. The remaining 25 items had a CVI score between 0.80 and 1.00. The 7 items that were removed had a low Content Validity Index and did not achieve sufficient consensus among the experts regarding their relevance to the topic, and therefore were not considered to represent more comprehensive and valid criteria. Based on this information, it can be said that the scale items are sufficient to measure the intended construct [8].

### Construct validity

Exploratory and Confirmatory Factor Analyses were applied to determine the construct validity. To assess whether the dataset was suitable for analysis, the KMO, Bartlett's Test of Sphericity, and Anti-Image values were checked. The KMO value obtained at this stage was found to be 0.971, which is above 0.60, indicating that the dataset is adequate for analysis [8, 25]. When determining the structure, it was emphasized that the difference between the factor loadings should be greater than 0.10 to prevent the presence of redundant items. During the analysis, the removal of 7 items indicated that the scale provided sufficient distinction for each factor. The two dimensions obtained were also supported by eigenvalues and the Scree plots graph, which contributed to strengthening the construct validity. The results of the confirmatory factor analysis showed that the fit indices were within acceptable limits. It was noted that low factor loadings (below 0.30) could pose issues for the scale's validity. However, in our study, the lowest factor loading was determined to be 0.67, which demonstrates that the scale exhibits a robust structure [28].

### Reliability analysis

For a measurement tool to be used, it must be reliable [28]. In our study, various methods were used to determine reliability, and the Cronbach Alpha and McDonald Omega values obtained from these methods were above 0.60, which makes the scale reliable. Particularly, the Cronbach alpha value of 0.952 obtained for the"Cognitive Distortions"subdimension demonstrates that this subdimension is highly reliable as an independent structure. Furthermore, the 0.925 value found for the"Cognitive Triad"dimension provides similar results. Reliability analysis was also supported by examining the differences in the means between the lower 27% and upper 27% groups. The statistically significant differences in these groups clearly indicate the reliability of the proposed structures. It was observed that there was a very good relationship with the parallel forms method, and criterion validity was achieved.

### Originality

This research aims to fill a significant gap in the literature by quantifying the cognitive mechanisms underlying stress caused by sleep disorders. The findings support the validity of Beck's cognitive theory in explaining the sleep-stress interaction and offer meaningful implications for clinical and research practice. In this context, the lack of a scale that directly measures the effects of sleep disorders on stress highlights an area that has been overlooked in the literature.

In the international literature, when examining studies related to the topic, no measurement tool has been found that directly measures the effect of sleep disorders on stress [7, 27, 34].The scales developed in various studies typically assess sleep quality or disorders, but they do not focus on measuring the impact of sleep disorders on stress levels. For example, the"COPD and Asthma Sleep Impact Scale (*CASIS*)"developed by Porkrzywinski et al. [26] is a tool designed to assess sleep disorders in individuals with COPD and asthma, but it does not measure the effect of sleep disorders on stress levels [26]. Similarly, the"Postpartum Sleep Quality Scale"developed by Yang et al. [34] aims to measure sleep quality in postpartum women but does not observe the effects of sleep disorders on stress [34]. The"Cumhuriyet Subjective Sleep Quality Scale"developed by Sarıçam [27] also only assesses sleep quality and does not measure the relationship between sleep disorders and stress [27]. The"Hospital-Acquired Insomnia Scale"developed by Çiftçi et al. [7] focuses on the causes of insomnia experienced during hospitalization but does not examine the effect of sleep disorders on stress levels [7]. None of these scales are designed to directly measure the effect of sleep disorders on an individual's stress levels.

In addition, there are various measurement tools that assess different psychological aspects related to sleep and stress. For example, the"Meta-Cognition Questionnaire-Insomnia"developed by Waine et al. [32] is used to measure distressing thoughts and beliefs specific to insomnia,however, this tool does not assess the effect of sleep disorders on stress [32].

Other scales in the international literature focus on measuring different sources of stress, but none specifically examine the direct relationship between sleep disorders and stress. For example, the"Chinese Parenting Stress Scale for Preschoolers'Parents"developed by Zhao et al. [36] measures the stress levels of parents of preschoolers but does not address the impact of sleep disorders on this stress [36]. Similarly, the"Maternal Postpartum Stress Scale"developed by Rados et al. [24] evaluates the stress levels of mothers in the postpartum period but does not investigate the stress levels associated with sleep disorders [24]. Likewise, the"Digital Stress Scale"developed by Xie et al. [33] is a tool for measuring digital stress and does not assess the relationship between sleep disorders and stress [33].

This literature review shows that there is no tool in the international literature that measures the stress levels caused by sleep disorders. In this regard, the scale we have developed makes a significant contribution to the literature as a unique tool that measures stress caused by sleep disorders in a unidimensional manner.

1. Neurocognitive foundations of the sleep-stress cycle
  The high reliability (*α* = 0.925) of the"Cognitive Triad"subscale confirms the distortions created by sleep deprivation in individuals'self-perception, future expectations, and environmental interpretation processes. These findings are consistent with neurocognitive models that explain how emotional regulation disorders, associated with reduced prefrontal cortex activity [13], lead to the chronicization of stress. For example, the high factor loadings in items such as"When my hope for the future decreases"support the hypothesis that sleep disorders trigger the learned helplessness cycle by inhibiting hippocampal neurogenesis [22]. This situation emphasizes that the sleep-stress relationship should not be considered as a one-way process, but rather as a synergistic cycle.
2. The mediating role of cognitive distortions and clinical ımplications
  The high internal consistency (*α* = 0.952) of the"Catastrophizing"subscale demonstrates that the irrational beliefs predicted by Beck's theory exacerbate the clinical picture. Especially in organically based disorders like obstructive sleep apnea, patients'automatic thoughts such as"Everything is going to get worse"increase cortisol secretion, fueling the physiological stress response [5].These findings suggest that psychoeducational programs should focus not only on sleep hygiene but also on restructuring cognitive distortions. In this context, it is foreseeable that the scale could be used as a monitoring tool to evaluate the effectiveness of CBT interventions.
3. Interdisciplinary contribution potential and limitations
  Integrating the scale with neuroimaging studies could reveal the relationships between amygdala-hippocampal activity and cognitive distortion scores. However, the limitations of the study's sample (only outpatient patients) restrict its generalizability to the broader population. Additionally, since 78% of the items are based on distortion types from Western literature, it may face criticism for not adequately reflecting"sleep-related myths"specific to Turkish culture (for example, the fear of"dying early if I sleep too little"). To overcome these limitations, community-based studies and cultural adaptation processes are recommended.
4. In-depth analysis of the bidirectional nature of the sleep-stress relationship
  While it is well-established in the literature that stress can both be a cause and a consequence of sleep disorders [14], this study particularly highlights the critical role of cognitive distortions in this cycle. For example, a patient with sleep apnea who experiences the anxiety of"I won’t be able to breathe"increases sympathetic nervous system activation, which exacerbates sleep fragmentation, in turn elevating stress hormones further. This pathological cycle can be quantified through the combined analysis of the scale's"Cognitive Triad"and"Distortions"dimensions. However, due to the cross-sectional design, the inability to clarify causality remains a fundamental limitation in the interpretation of the findings.
  This scale suggests that sleep-related stress should be addressed within a new framework called the Cognitive Triad + Cognitive Distortions Model. This model would allow clinicians not only to assess the severity of symptoms but also to map the underlying mental processes. Future studies should explore the integration of the scale with digital therapeutic applications to test its real-time data collection capacity.

## Conclusıon and recommendatıons

This study suggests that stress resulting from sleep disorders should be addressed within a new framework called the"Cognitive Triad + Cognitive Distortions"model. This scale provides clinicians with the ability to systematically map not only the symptom severity of patients but also the underlying cognitive processes (self-perception, future expectations, and cognitive distortions related to world/environment interpretation).

In clinical applications, it is recommended that the scale be used as a screening tool in sleep clinics, with early interventions, such as cognitive-behavioral therapy (CBT), being initiated for patients scoring high. Additionally, the development of modules specifically targeting the"catastrophizing"subdimension in psychoeducation programs may play a critical role in restructuring irrational thoughts that lead to increased cortisol levels.

Research-focused recommendations include integrating the scale with digital therapeutic applications to test its real-time data collection capacity and examining the relationships between amygdala-hippocampal activity and distortion scores using neuroimaging techniques.

In conclusion, this scale stands out as a tool that can contribute to personalized treatment planning in clinical practice by quantifying the bidirectional relationship between stress and sleep disorders, and facilitate the testing of new hypotheses in interdisciplinary research. The potential for integration with digital health technologies may allow the scale to be used for dynamic data analysis in future studies.

## Data Availability

No datasets were generated or analysed during the current study.

## Abbreviations

α: Cronbach’s alpha
CFA: Confirmatory factor analysis
CMIN/DF: Chi-square/df ratio
CFI: Comparative fit index
EFA: Exploratory factor analysis
IFI: Incremental fit index
NFI: Normed fit index
PSS: Perceived stress scale
PGFI: Parsimonious goodness-of-fit index
PNFI: Parsimony normed fit index
PSQI: Pittsburgh sleep quality index
RMSEA: Root mean square error of approximation
RMR: Root mean square residual
SESDS: Scale on the effects of sleep disorder on stress
SRMR: Standardized root mean square residual
TLI: Tucker-Lewis index
VAS: Visual analog scale
ω: McDonald’s omega
